# A Real-World Study on the Effectiveness and Safety of Pembrolizumab Plus Chemotherapy for Nonsquamous NSCLC

**DOI:** 10.1016/j.jtocrr.2021.100265

**Published:** 2021-12-16

**Authors:** Daichi Fujimoto, Satoru Miura, Kenichi Yoshimura, Kazushige Wakuda, Yuko Oya, Koji Haratani, Shoichi Itoh, Takehiro Uemura, Ryotaro Morinaga, Takayuki Takahama, Kazuhisa Nakashima, Motoko Tachihara, Go Saito, Junko Tanizaki, Kohei Otsubo, Satoshi Ikeda, Hirotaka Matsumoto, Satoshi Hara, Akito Hata, Takeshi Masuda, Nobuyuki Yamamoto

**Affiliations:** a Internal Medicine III, Wakayama Medical University, Wakayama, Japan; b Department of Respiratory Medicine, Kobe City Medical Center General Hospital, Kobe, Japan; c Department of Internal Medicine, Niigata Cancer Center Hospital, Niigata, Japan; d Medical Center for Translational and Clinical Research, Hiroshima University Hospital, Hiroshima University, Hiroshima, Japan; e Division of Thoracic Oncology, Shizuoka Cancer Center, Shizuoka, Japan; f Department of Thoracic Oncology, Aichi Cancer Center Hospital, Nagoya, Japan; g Department of Medical Oncology, Faculty of Medicine, Kindai University, Osaka-Sayama, Japan; h Department of Thoracic Oncology, Hyogo Cancer Center, Akashi, Japan; i Department of Respiratory Medicine, Allergy and Clinical Immunology, Nagoya City University School of Medical Sciences, Nagoya, Japan; j Department of Thoracic Medical Oncology, Oita Prefectural Hospital, Oita, Japan; k Department of Medical Oncology, Kindai Nara Hospital, Ikoma, Japan; l Department of Internal Medicine, Division of Medical Oncology & Respiratory Medicine, Shimane University Faculty of Medicine; m Division of Respiratory Medicine, Department of Internal Medicine, Kobe University Graduate School of Medicine, Kobe, Japan; n Department of Respirology, Chiba University Graduate School of Medicine, Chiba, Japan; o Department of Medical Oncology, Kishiwada City Hospital, Kishiwada, Japan; p Department of Respiratory Medicine, Kitakyushu Municipal Medical Center, Kitakyushu, Japan; q Department of Respiratory Medicine, Kanagawa Cardiovascular and Respiratory Center, Yokohama, Japan; r Department of Respiratory Medicine, Hyogo Prefectural Amagasaki General Medical Center, Amagasaki, Japan; s Department of Respiratory Medicine, Itami City Hospital, Itami, Japan; t Department of Medical Oncology, Kobe Minimally Invasive Cancer Center, Kobe, Japan; u Department of Respiratory Internal Medicine, Hiroshima University Hospital, Hiroshima, Japan

**Keywords:** Immune checkpoint inhibitor, Pembrolizumab, Pneumonitis, Programmed Death-1, Programmed Death-Ligand 1

## Abstract

**Introduction:**

The real-world effectiveness of combination treatment with cytotoxic chemotherapy and programmed cell death protein-1 or programmed death-ligand 1 inhibitor for NSCLC, especially for the elderly (aged ≥75 y) or those with poor performance status (≥2), has not been fully elucidated. We investigated the real-world effectiveness and safety of this combination therapy in these populations.

**Methods:**

This multicenter retrospective study evaluated patients who are chemo-naïve with advanced NSCLC who received a combination of platinum, pemetrexed, and pembrolizumab between December 2018 and June 2019. This was an updated prespecified secondary analysis with the primary objective of investigating the safety and effectiveness in this cohort.

**Results:**

Overall, 299 patients were included. Multivariate analysis identified performance status (0–1) and programmed death-ligand 1 tumor proportion score (≥50%) as significant independent predictors of progression-free survival (*p* = 0.007, and *p* = 0.003, respectively). The incidence of severe adverse events (AEs) was higher in the elderly and those with poor performance status than in their younger and good performance status counterparts. A total of 71 patients developed AEs that led to treatment discontinuation, and AE-related treatment discontinuation occurred at a significantly higher rate in older patients (median [range]) (70 [46–82] y) than in younger patients (68 [31–84] y) (*p* <0.001).

**Conclusions:**

Combination treatment with pembrolizumab plus chemotherapy had low real-world effectiveness for poor performance status patients. Severe AEs occurred at a higher rate in the elderly and poor performance status patients, and the AE-related treatment discontinuation rate increased with age. Physicians should be cautious about using this regimen, especially in the elderly and poor performance status patients.

## Introduction

Lung cancer is the leading cause of cancer-related deaths worldwide.^1^ NSCLC accounts for approximately 80% of all lung cancers, and most NSCLC cases are unresectable and metastatic at initial diagnosis.^2^ The development of immune checkpoint inhibitors, such as programmed cell death protein-1 (PD-1) and programmed death-ligand 1 (PD-L1), has markedly changed the treatment strategy for NSCLC. The addition of the PD-1 inhibitor, pembrolizumab, to the combination of a platinum agent and pemetrexed has recently become a standard first-line treatment for patients with previously untreated metastatic nonsquamous NSCLC without driver oncogenes.^3^

The eligibility criteria in recent clinical trials have become more stringent to establish treatment efficacy.^4^^,^^5^ Therefore, only a few patients in a relatively good general condition without organ failure meet the eligibility criteria for clinical trials.^4^^,^^5^ As such, the outcomes of clinical trials are not entirely representative of those in real-world patients. Efficacy is investigated in these ideal settings to minimize potential bias affecting the internal validity of an intervention’s effects on the outcome through randomization and stratification. Alternatively, effectiveness refers to the treatment performance in a real-world setting with high external validity through observational studies.^6^ Specifically, there is scarce evidence regarding the safety and effectiveness of combination therapy in underrepresented populations, such as the elderly or those with poor performance status (PS). In participants aged more than or equal to 75 years in the KEYNOTE-189 trial,^7^ a trend toward lower effectiveness of chemotherapy plus pembrolizumab was noted with an apparent detrimental effect (hazard ratio [HR]: progression-free survival [PFS], 1.73 [95% confidence interval (CI): 0.77–3.90]; overall survival [OS], 2.09 [95% CI: 0.84–5.23]).

PD-1 axis inhibitors can clinically cause inflammatory side effects (i.e., immune-related adverse events [irAEs]) that differ from those related to conventional systemic therapy. Severe irAEs are problematic because they can lead to difficulties in subsequent therapy and be potentially life-threatening.^8^^,^^9^ The feasibility of the combination of a platinum agent, pemetrexed, and pembrolizumab was shown in the KEYNOTE-189 trial.^3^ However, the frequency of adverse events (AEs) tended to be higher in patients receiving combination therapy than in those receiving pembrolizumab monotherapy or platinum-doublet chemotherapy. Furthermore, previous studies revealed that the rate of AEs was higher in a real-world population than in previous clinical trials.^10^^,^^11^ More AEs occurred in the elderly or patients with poor PS owing to their co-morbidities and lower physiological function.

In this study, we investigated the effectiveness and safety of combination therapy of cytotoxic chemotherapy and pembrolizumab in patients with previously untreated nonsquamous NSCLC in a real-world setting.

## Materials and Methods

### Study Design and Patients

This was a multicenter, retrospective, hospital-based cohort study of consecutive patients with chemotherapy-naïve advanced NSCLC who received combination therapy at any of the 36 hospitals in Japan between December 2018 and June 2019. Clinical data for each patient were extracted from medical charts and entered into a database.

This report is an updated prespecified secondary analysis with the primary objective of investigating the safety and effectiveness in this cohort. The primary analysis aimed to investigate the incidence of pneumonitis within 90 days of initiating combination therapy, and the results were reported in a previous study.^12^ The cutoff date for data collection in this study was April 30, 2020. The cutoff date for data collection in the primary analysis was October 1, 2019.

Patients aged more than 20 years were enrolled if they had pathologically confirmed metastatic nonsquamous NSCLC without sensitizing EGFR mutations or anaplastic lymphoma kinase rearrangements and received a combination of platinum, pemetrexed, and pembrolizumab (combination therapy) as first-line treatment.

The study design was approved by the ethical institutional review board of each participating institution. The requirement for written informed consent was waived owing to the retrospective nature of the study.

### Definitions and Assessments

Smoking status was categorized as never (i.e., never smoked), current (i.e., smoked within 1 y of diagnosis), and former (i.e., other smoking status). PD-L1 expression was assessed using the PD-L1 immunohistochemistry 22C3 pharmDx assay and was categorized by the tumor proportion score (TPS). The presence of pre-existing interstitial lung disease and emphysema was determined by the treating pulmonologist or oncologist on the basis of computed tomography images before the start of combination therapy. The elderly population was defined as those aged more than or equal to 75 years, and poor PS was defined as an Eastern Cooperative Oncology Group PS of ≥2.

Clinical staging was performed according to the TNM classification (eighth edition). Antitumor responses were assessed according to the Response Evaluation Criteria in Solid Tumors (version 1.1) by the investigators of the included institutions. PFS and OS were calculated as the interval between the date of commencing combination therapy and the date of disease progression or death from any cause or the date of death from any cause, respectively.

### Safety Analysis

AEs were evaluated by the attending physician according to the Common Terminology Criteria for Adverse Events (version 5.0). Safety was investigated using AE data related to combination therapy, including all-grade pneumonitis, nephrotoxicity, grade greater than or equal to 3 nonhematologic AEs, and grade greater than or equal to 4 hematologic AEs. Severe AEs were defined as febrile neutropenia and grade greater than or equal to 3 nonhematologic AEs. Pneumonitis and nephrotoxicity were defined as AEs of special interest because of their high incidence in previous clinical trials^3^^,^^13^ using the same treatment. The diagnosis and grade of pneumonitis were determined by the treating pulmonologist or the oncologist on the basis of the clinical and radiographic parameters and the exclusion of alternative etiologies (e.g., congestive heart failure, infection, and tumor progression).

### Statistical Analyses

Age was compared using the Wilcoxon rank-sum test. Dichotomous variables were analyzed using the chi-square test or Fisher’s exact test, as appropriate. The Kaplan-Meier method was used to estimate survival outcomes. To determine the associations between patient characteristics and survival outcomes, a multivariate Cox proportional hazards model was developed for all clinically important factors (age, sex, smoking status, Eastern Cooperative Oncology Group PS, and PD-L1 status) identified on the basis of previous studies of immune checkpoint inhibitors.^3^^,^^8^ The results are expressed as HRs with 95% CIs. A two-sided *p* value less than 0.05 was considered statistically significant.

## Results

### Patient Characteristics and Outcomes

A total of 299 patients were enrolled in this study. Patient characteristics are summarized in Table 1. The median age was 68.0 years, and there were 43 elderly patients (14%). Most patients were men (74%), had a history of smoking (84%), had a PS of 0 to 1 (95%), and had adenocarcinoma histology (93%). The PD-L1 TPS was <1%, 1% to 49%, ≥50%, and not investigated in 37%, 35%, 21%, and 6% of patients, respectively. The total objective response rate was 50% (2% of patients achieved a complete response; 48% achieved a partial response). Overall, 33% of patients had stable disease, 13% had progressive disease, and 4% had not been evaluated.

**Table 1:** Baseline Patient Characteristics

Characteristics	Patients (N = 299)
Age (y)	
Median (range)	68.0 (31–84)
Sex, n (%)	
Male	222 (74)
Smoking status, n (%)	
Current	116 (39)
Former	134 (45)
Never	49 (16)
ECOG PS, n (%)	
0	95 (32)
1	190 (64)
2	11 (4)
3	3 (1)
Histologic diagnosis, n (%)	
Adenocarcinoma	278 (93)
Others	21 (7)
Stage, n (%)	
3	11 (4)
4	228 (76)
Recurrence after surgery	46 (15)
Recurrence after radiotherapy	14 (5)
PD-L1 TPS, n (%)	
≥50%	65 (21)
1%–49%	104 (35)
<1%	112 (37)
Not investigated	18 (6)
Pre-existing interstitial lung disease, n (%)	13 (4)
Emphysema, n. (%)	114 (38)
Previous thoracic radiotherapy, n (%)	33 (11)

ECOG PS, Eastern Cooperative Oncology Group performance status; PD-L1, programmed death-ligand 1; TPS, tumor proportion score.

^a^Smokers versus never-smokers and greater than or equal to 50% versus less than 50% PD-L1 expression.

### Effectiveness in the Overall Population

During a median follow-up of 11.7 (interquartile range: 9.8–13.6) months, 194 PFS events (65%) and 81 OS events (27%) were observed. The median PFS and OS were 8.6 (95% CI: 8.6–9.5) months and not reached (NR) (95% CI: 15.7–NR), respectively (see Supplementary Fig. 1 in Supplementary Data 1, which shows the survival curves). Multivariate analysis identified a PS of 0 to 1 (HR = 0.37, 95% CI: 0.21–0.74, *p* = 0.007) and PD-L1 TPS of greater than or equal to 50% (HR = 0.57, 95% CI: 0.38–0.83, *p* = 0.003) as significant independent predictors of PFS (Table 2) (see Supplementary Fig. 2 in Supplementary Data 2, which shows the Kaplan-Meier curves for PFS and OS stratified by PD-L1 status).

**Table 2:** Multivariate Analysis of Progression-Free Survival

Characteristics	Progression-Free Survival	
	HR (95% CI)	*p* Value
Age (≥75 vs. <75 y)	1.18 (0.77–1.74)	0.428
Sex (male vs*.* female)	1.37 (0.91–2.10)	0.137
Smoking status (never vs. current or former smoker)	1.34 (0.82–2.16)	0.246
ECOG PS (0–1 vs. 2–3)	0.37 (0.21–0.74)	0.007
PD-L1 expression (≥50% vs. <50%)	0.57 (0.38–0.83)	0.003

CI, confidence interval; ECOG PS, Eastern Cooperative Oncology Group performance status; HR, hazard ratio; PD-L1, programmed death-ligand 1.

### Safety in the Overall Population

Overall, 57 patients (19%) had grade greater than or equal to 3 nonhematologic AEs, and 19 patients (6%) had grade greater than or equal to 4 hematologic AEs (see Table in Supplementary Data 3, which shows the treatment-related AEs). A total of 10 patients (3.3%) died of treatment-related AEs attributed to combination therapy: pneumonitis (n = 4), febrile neutropenia (n = 2), sepsis (n = 2), lung infection (n = 1), and sudden death not otherwise specified (n = 1). Among the patients who developed grade greater than or equal to 3 nonhematologic AEs, the most frequent AE was pneumonitis (5.0% of all patients). There was no significant difference in patient characteristics between those with and without severe AEs (Table 3).

**Table 3:** Comparison Between Patients With and Without Severe AEs

Characteristics	Patients With Severe AEs (n = 60)	Patients Without Severe AEs (n = 239)	*p* Value
Age (y)			0.21
Median (range)	68 (46–80)	68 (31–84)	
Sex, n (%)			0.88
Male	45 (75)	177 (74)	
Smoking status, n (%)			0.85^a^
Current	24 (40)	92 (38)	
Former	27 (45)	107 (45)	
Never	9 (15)	40 (17)	
ECOG PS, n (%)			0.17^a^
0	12 (20)	83 (35)	
1	43 (72)	147 (61)	
2	3 (5)	8 (3)	
3	2 (3)	1 (1)	
Histology, n (%)			0.15
Adenocarcinoma	53 (88)	225 (94)	
Others	7 (12)	14 (6)	
Stage, n (%)			
3	2 (3)	9 (4)	
4	51 (85)	177 (74)	
Recurrence after surgery	7 (12)	39 (16)	
Recurrence after radiotherapy	0 (0)	14 (6)	
PD-L1 TPS, n (%)			0.86^a^
≥50%	13 (22)	52 (22)	
1%–49%	22 (37)	82 (34)	
<1%	19 (32)	93 (39)	
Not investigated	6 (10)	12 (5)	
Pre-existing interstitial lung disease, n (%)	3 (5)	10 (4)	0.73
Emphysema, n (%)	25 (42)	89 (37)	0.53
Previous thoracic radiotherapy, n (%)	6 (10)	27 (11)	1.00

AE, adverse event; ECOG PS, Eastern Cooperative Oncology Group performance status; PD-L1, programmed death-ligand 1; TPS, tumor proportion score.

a Smokers versus never-smokers, ECOG PS 0 to 1 versus ECOG PS 2 to 3, and greater than or equal to 50% versus less than 50% PD-L1 expression.

Throughout the follow-up period, 71 patients (24%) discontinued all-treatment components owing to AEs; of them, 39 (55%) had pneumonitis. Treatment was discontinued in the induction and maintenance phases in 37 (52%) and 34 patients (48%), respectively. Pneumonitis was the most frequent AE leading to all-treatment discontinuation in the induction (n = 16) and maintenance (n = 21) phases. A comparison of the characteristics between patients with and without AE-related discontinuation of all-treatment components is shown in Table 4. AE-related treatment discontinuation occurred at a significantly higher rate in older patients than in younger patients (median [range]) (70 [46–82] versus 68 [31–84], respectively, *p* <0.001).

**Table 4:** Comparison Between Patients With and Without Toxicity-Related Discontinuation of All-Treatment Components

Characteristics	Patients With Discontinuation (n = 71)	Patients Without Discontinuation (n = 228)	*p* Value
Age (y)			<0.001
Median (range)	70 (46–82)	68 (31–84)	
Sex, n (%)			0.30
Male	56 (79)	166 (73)	
Smoking status, n (%)			0.54^a^
Current	29 (41)	87 (38)	
Former	32 (45)	102 (45)	
Never	10 (14)	39 (17)	
ECOG PS, n (%)			0.83^a^
0	21 (30)	74 (32)	
1	47 (66)	143 (63)	
2	1 (1)	10 (4)	
3	2 (3)	1 (1)	
Histology, n (%)			0.60
Adenocarcinoma	65 (92)	213 (93)	
Others	6 (8)	15 (7)	
Stage, n (%)			
3	3 (4)	8 (4)	
4	55 (77)	173 (76)	
Recurrence after surgery	11 (16)	35 (15)	
Recurrence after radiotherapy	2 (3)	12 (5)	
PD-L1 TPS, n (%)			0.95^a^
≥50%	15 (21)	50 (22)	
1%–49%	22 (31)	82 (36)	
<1%	27 (38)	85 (37)	
Not investigated	7 (10)	11 (5)	
Pre-existing interstitial lung disease, n (%)	5 (7)	8 (4)	0.23
Emphysema, n (%)	30 (42)	84 (37)	0.41
Previous thoracic radiotherapy, n (%)	10 (14)	23 (10)	0.36

ECOG PS, Eastern Cooperative Oncology Group performance status; PD-L1, programmed death-ligand 1; TPS, tumor proportion score.

a Smokers versus never-smokers, ECOG PS 0 to 1 versus ECOG PS 2 to 3, and greater than or equal to 50% versus less than 50% PD-L1 expression.

### AEs of Special Interest

There were 54 patients (18%) with all-grade pneumonitis and 15 patients (5.0%) with grade greater than or equal to 3 pneumonitis. Whereas 61 patients (20%) had all-grade nephrotoxicity, and three patients (1.0%) had grade greater than or equal to 3 nephrotoxicity. The median time to pneumonitis onset from the start of combination therapy was 4.0 (interquartile range: 1.9–5.7) months. Among the patients who developed pneumonitis, almost all (n = 39; 72%) discontinued treatment owing to AEs. The median time to nephrotoxicity onset from the start of combination therapy was 2.1 (interquartile range: 0.3–4.2) months. Among the patients with nephrotoxicity, five (8.2%) discontinued therapy owing to AEs (see Table in Supplementary Data 4, which shows the severity of pneumonitis and nephrotoxicity).

### Effectiveness in the Elderly and Populations with Poor PS

**Figure 1:**
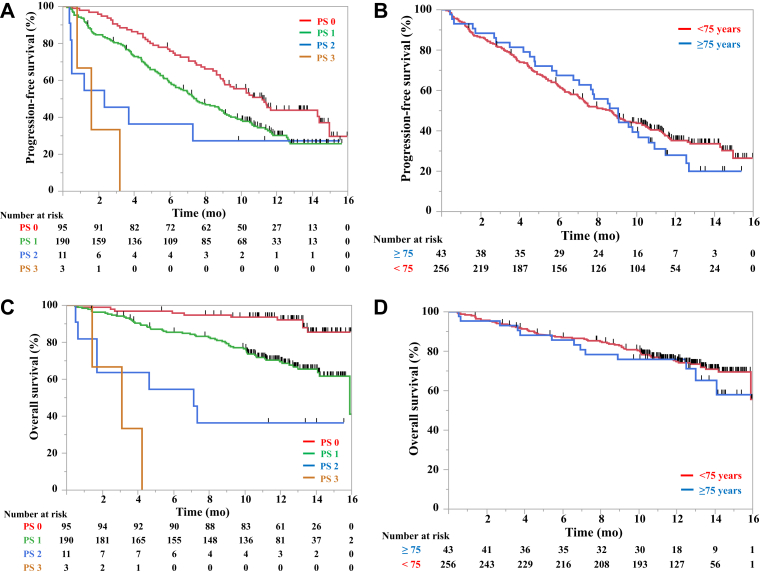
Kaplan-Meier curves of survival. Progression-free survival curves stratified by (*A*) PS and (*B*) age and overall survival curves stratified by (*C*) PS and (*D*) age. PS, performance status.

As issues related to effectiveness and safety were concerning in this cohort, further analysis of PS or age was performed. The Kaplan-Meier curves for PFS and OS stratified by these factors are illustrated in Figure 1. The median PFS of those aged less than 75 and more than or equal to 75 years was 8.5 (95% CI: 7.0–9.9) and 8.9 (95% CI: 6.7–10.5), respectively. The median OS of those aged less than 75 and greater than or equal to 75 years was NR (95% CI: 15.7–NR) and NR (95% CI: 12.8–NR), respectively. Whereas the median PFS of patients with a PS of 0, 1, 2, and 3 was 11.0 (95% CI: 8.9–14.2), 7.4 (95% CI: 6.1–8.9), 2.3 (95% CI: 0.4–NR), and 1.6 (95% CI: 0.8–3.2), respectively. The median OS of patients with a PS of 0, 1, 2, and 3 was NR, 15.7 (95% CI: 15.7–NR), 7.0 (95% CI: 0.6–NR), and 3.1 (95% CI: 1.4–4.2), respectively.

### Safety in the Elderly and Populations with Poor PS

The rate of severe AEs was higher in the elderly than in younger patients (26% versus 19%, respectively, *p* = 0.312), although the difference was not significant. The rate of AE-related discontinuation of all-treatment components was also significantly higher in the elderly (40% versus 21%, respectively, *p* = 0.012). We performed further safety analysis on the basis of the following three age categories: less than 65, 65 to 74, and more than or equal to 75 years (see Table in Supplementary Data 5, which shows the patient characteristics and safety profiles stratified by age). Higher rates of severe AEs (16%, 21%, and 26%, respectively) and AE-related discontinuation of all-treatment components (14%, 27%, and 40%, respectively) were observed in the elderly.

The rates of severe AEs in those with a PS of 0, 1, 2, and 3 were 13%, 23%, 27%, and 67%, respectively. Safety analysis by PS also revealed higher rates of severe AEs in those with poor PS (2–3) than in those with good PS (0–1), with no significant difference (36% versus 19%, respectively, *p* = 0.166). The incidence rates of AE-related discontinuation of all-treatment components were similar between those with good and poor PS (24% versus 21%, respectively, *p* = 1.00).

Among the 10 patients who died of combination treatment-related AEs, two were elderly (2 of 43; 4.7%), two had poor PS (2 of 14; 14%), and six were younger and had good PS (6 of 242; 2.5%).

## Discussion

This study presents data on the safety and effectiveness of the combination of platinum, pemetrexed, and pembrolizumab in a real-world setting. This combination treatment had low real-world effectiveness for patients with poor PS. As for safety, treatment-related AEs, particularly pneumonitis and nephrotoxicity, occurred at a higher rate. In addition, severe AEs occurred at a higher rate in the elderly and patients with poor PS, and the rate of AE-related treatment discontinuation increased with age.

The real-world PFS of the combination of platinum, pemetrexed, and pembrolizumab in this analysis is similar to that in a clinical trial^3^ of combination treatment for NSCLC (median = 8.8 mo). However, the rate of treatment-related AEs, particularly pneumonitis and nephrotoxicity, was higher than that in a previous clinical trial.^3^ Consistent with the previous findings of clinical trials of PD-1 axis inhibitors for lung cancer, the most frequent severe irAE in our study was pneumonitis. All-grade and severe pneumonitis occurred in 18% and 5% of patients in our study, respectively. Despite a similar follow-up period, these rates are considerably higher than those in the KEYNOTE-189 trial,^3^ wherein the frequency of all-grade and severe pneumonitis was only 4.4% and 2.7%, respectively. We also observed a higher rate of nephrotoxicity and AE-related treatment discontinuation in our real-world population. In total, 24% of patients discontinued all-treatment components owing to toxicities, which is higher than the 14% reported in the KEYNOTE-189 trial.^3^ This difference may be due to the dissimilarities between patient populations in the real world and those in clinical trials. Thus, careful attention should be paid to differences between real-world and clinical trial settings when using this combination treatment.

Our cohort also provides important data regarding patient subgroups (the elderly and patients with poor PS) who are underrepresented in clinical trials. The results revealed that poor PS was a strong independent negative predictor of PFS, consistent with the finding that poor PS predicted PFS in patients with NSCLC receiving PD-1 axis inhibitors.^3^^,^^10^^,^^14^ Regarding the safety of this combination therapy according to PS, the rate of severe treatment-related AEs was higher in those with poor PS, with some patients dying owing to treatment-related AEs. Regarding age, severe treatment-related AEs and treatment discontinuation owing to AEs increased with age in our study. The rate of AE-related treatment discontinuation was approximately two times higher in the elderly than in younger patients in our study. Our data suggest that this combination therapy should be considered carefully for the elderly and populations with poor PS. Frailty is significantly correlated with age and PS and is associated with treatment-related toxicities and survival outcomes.^15^^,^^16^ A recent study^17^ reported that the plasma concentration of anticancer drugs is higher in frail patients owing to co-morbidities and reduced physiological function. Given the increasing number of elderly and frail patients, further studies are required to investigate treatment strategies with better risk-to-benefit and cost-to-benefit ratios for these patients.

In our study, we observed a higher rate of severe treatment-related AEs in those with poor PS. However, the incidence of AE-related discontinuation of all-treatment components were similar between those with good PS (0–1) and poor PS (2–3). In general, the higher the rate of severe toxicity, the higher the toxicity-related discontinuation rate. However, most patients with poor PS in our cohort developed progressive disease, whereas treatment was paused owing to severe toxicities. In these patients, treatment discontinuation was primarily owing to disease progression and not owing to AEs. This supported the conclusion that there was no significant correlation between the rate of severe toxicity and the rate of treatment discontinuation in our study.

Several relatively large real-world studies (n ≥100) on first-line chemo-immunotherapy were focused on patients with advanced NSCLC.^18^, ^19^, ^20^ A previous report revealed that the survival estimates were lower than those reported in pivotal clinical trials,^20^ whereas other reports have stated that the survival estimates may be as effective as in the clinical trial, in line with our findings.^18^^,^^19^ However, data regarding subsets underrepresented in clinical trials are scarce. A recent article included 25 elderly patients who received pemetrexed-based combination therapy and revealed that the PFS and OS rates of the elderly were significantly worse than those of the nonelderly.^18^ However, PD-L1 was not a predictive factor for PFS and OS in this study. Concerning safety, the discontinuation rate of treatment components tended to be higher, but not significantly, in the elderly than in the nonelderly patients in this study. These results of effectiveness were different from ours, but the results concerning safety had a similar trend. Although several previous studies of immunotherapy have shown that increased age was not associated with a higher irAE rate,^21^, ^22^, ^23^, ^24^ the clinical trials of cytotoxic agents for the elderly revealed a higher rate of AEs.^25^^,^^26^ In addition, a trend toward lower effectiveness of chemotherapy plus pembrolizumab was noted with an apparent detrimental effect in participants aged more than or equal to 75 years in the KEYNOTE-189 trial.^7^ On the basis of these results and those obtained from our study, the safety of chemo-immunotherapy for the elderly should be considered an important issue to investigate. As a recent prospective observational study revealed that the G8 screening identified a subgroup with a higher risk of AEs in the elderly,^27^ our findings supported the need for further research for the use of comprehensive geriatric assessment to identify the patients at high risks of developing AEs.

Our study had several limitations. First, it was a retrospective study. Therefore, the safety assessment in this study was limited to a severe grade or any grade of pneumonitis and nephrotoxicity, which are easier to judge objectively. Second, this study included a small number of patients with poor PS, and almost all of the patients in our cohort were of a single ethnicity (Japanese). However, to our knowledge, this study included the largest multicenter cohort of such patients evaluating the PFS rates and the reliability of this combination, thereby providing novel findings. Third, we could not perform a comprehensive geriatric assessment or evaluate the Charlson Comorbidity Index. The results of our study may serve as the basis for conducting future prospective studies using these factors to identify the patients at high risks of developing AEs among the elderly and those with poor PS who are underrepresented in clinical trials.

In conclusion, combination treatment with pembrolizumab plus chemotherapy has low real-world effectiveness in patients with NSCLC with poor PS. Treatment-related AEs, particularly pneumonitis and nephrotoxicity, occurred at a significantly higher rate in a real-world setting. In addition, severe AEs occurred at a higher rate in the elderly and patients with poor PS. Furthermore, the rate of AE-related treatment discontinuation increased with age. As such, physicians should be particularly cautious about using this regimen in the elderly and PS patients with poor PS.

## CRediT Authorship Contribution Statement

**Daichi Fujimoto:** Conceptualization, Data curation, Formal analysis, Funding acquisition, Investigation, Methodology, Project administration, Writing - original draft, Writing - review & editing.

**Satoru Miura:** Conceptualization, Data curation, Investigation, Methodology, Project administration, Writing - original draft, Writing - review & editing.

**Kenichi Yoshimura:** Formal analysis, Methodology, Writing - original draft, Writing - review & editing.

**Kazushige Wakuda, Yuko Oya, Shoichi Itoh, Takehiro Uemura, Ryotaro Morinaga, Takayuki Takahama, Kazuhisa Nakashima, Motoko Tachihara, Go Saito, Junko Tanizaki, Kohei Otsubo, Satoshi Ikeda, Hirotaka Matsumoto, Satoshi Hara, Akito Hata, Takeshi Masuda, Koji Haratani:** Data curation, Investigation, Writing - original draft, Writing - review & editing.

**Nobuyuki Yamamoto**: Funding acquisition, Project administration, Writing - original draft, Writing - review & editing.
