# Supplementary figures and images for: A Real-World Study on the Effectiveness and Safety of Pembrolizumab Plus Chemotherapy for Nonsquamous NSCLC
Source: JTO Clin Res Rep. 2021 Dec 16;3(2):100265. doi: 10.1016/j.jtocrr.2021.100265 (PMC8819387; doi:10.1016/j.jtocrr.2021.100265)

Supplementary Figure 1A

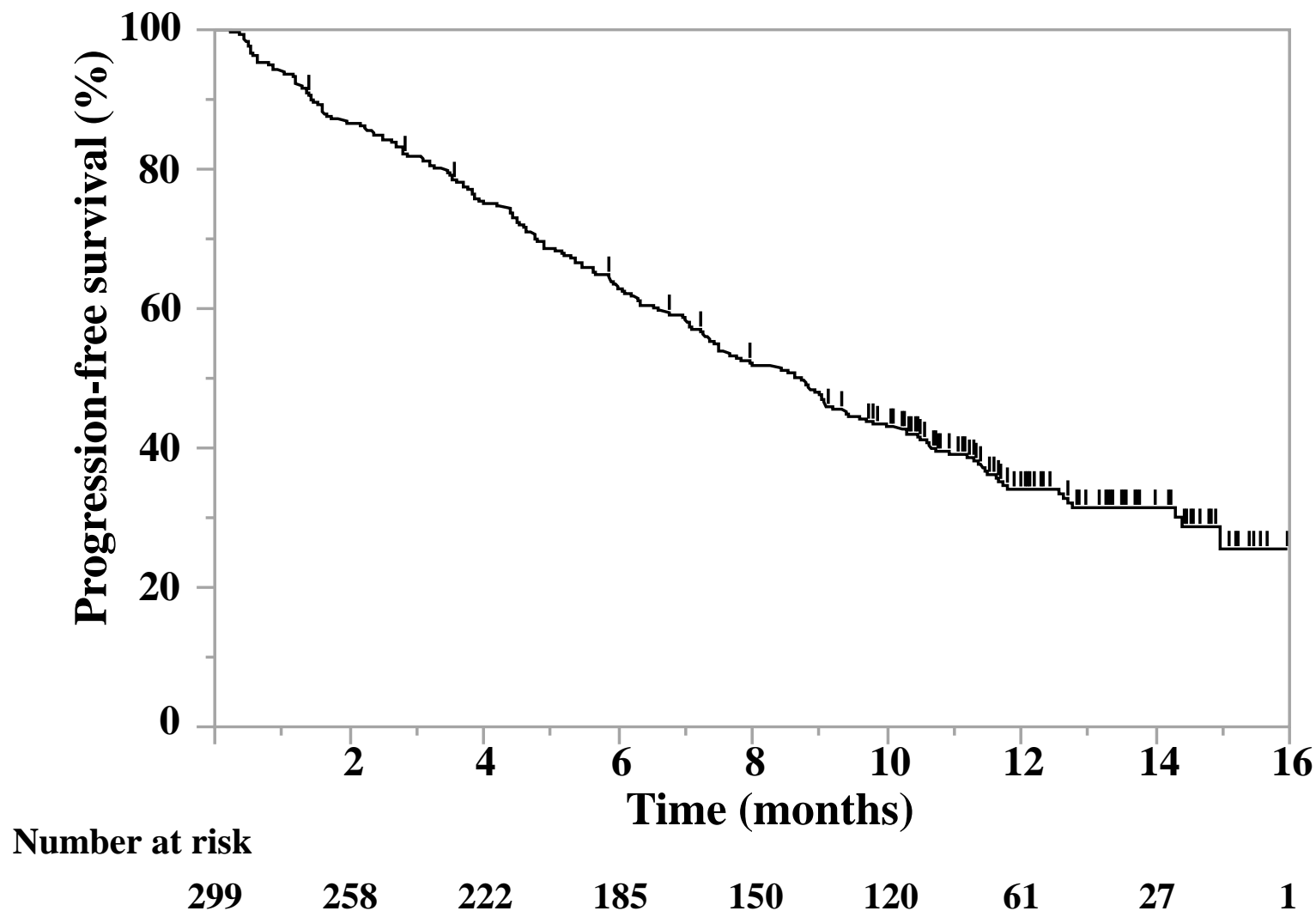

Supplementary Figure 1B

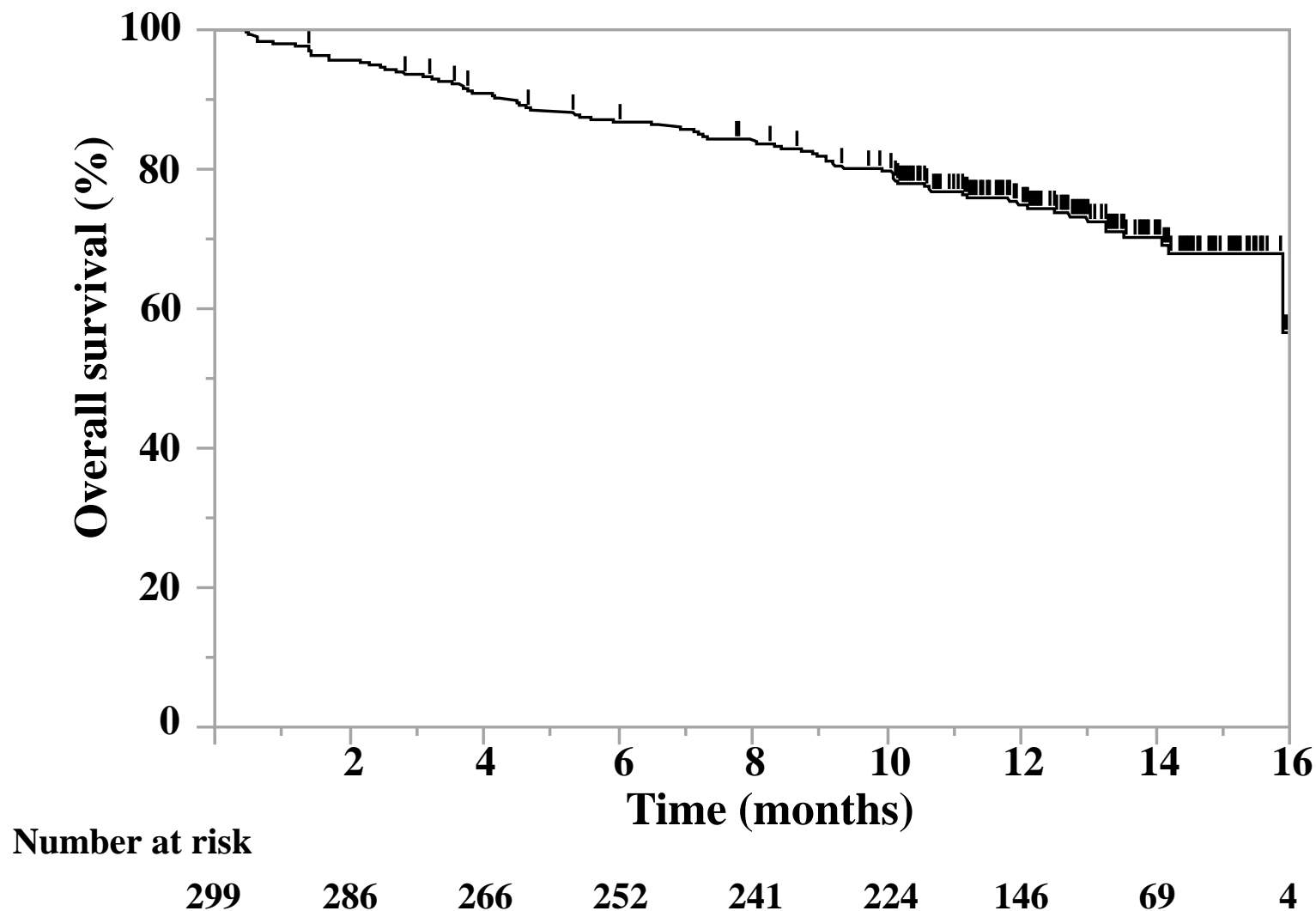

Supplement: Supplemental Data 1 [file mmc1.pdf]

Supplementary Figure 2A

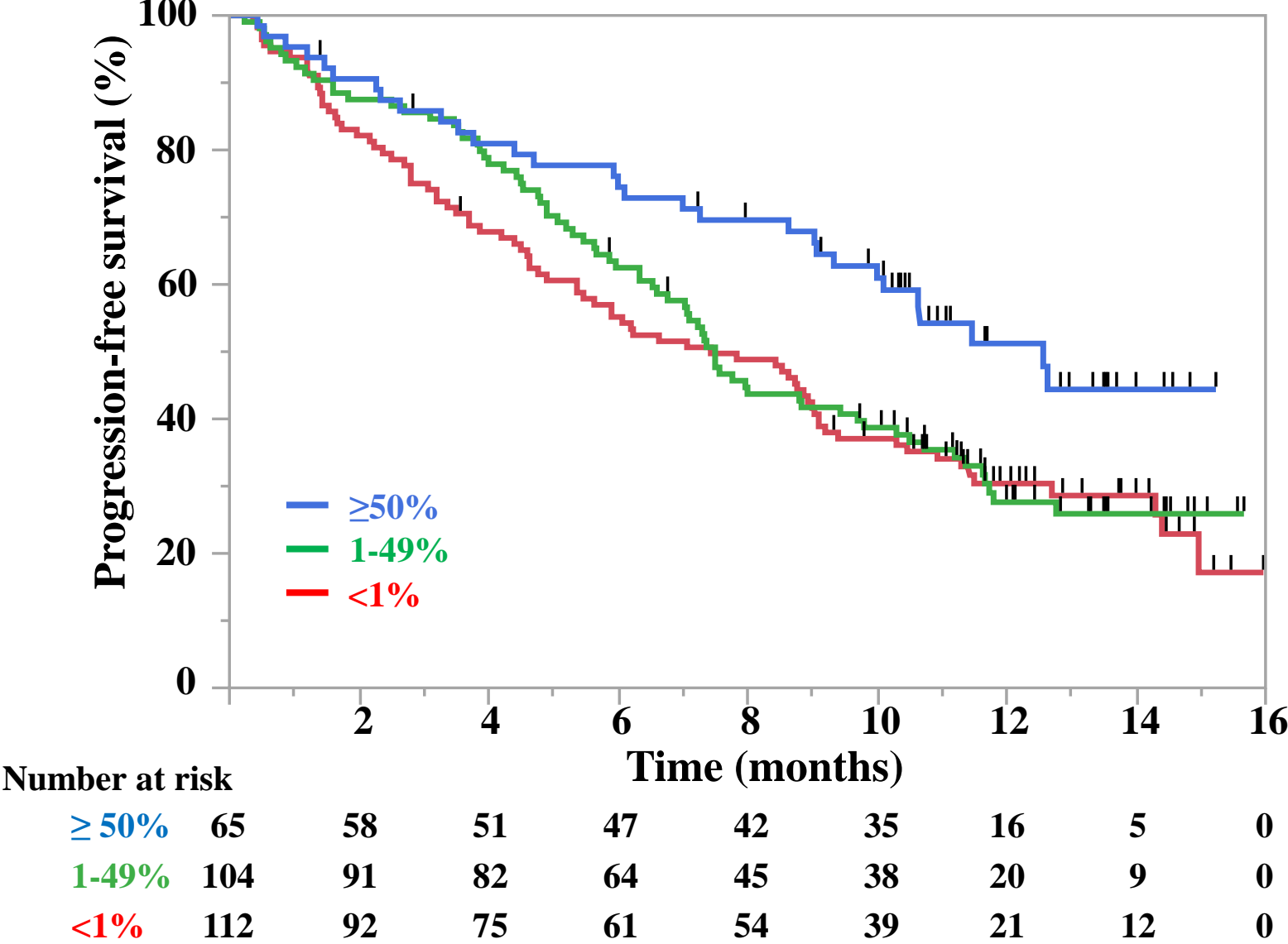

Supplementary Figure 2B

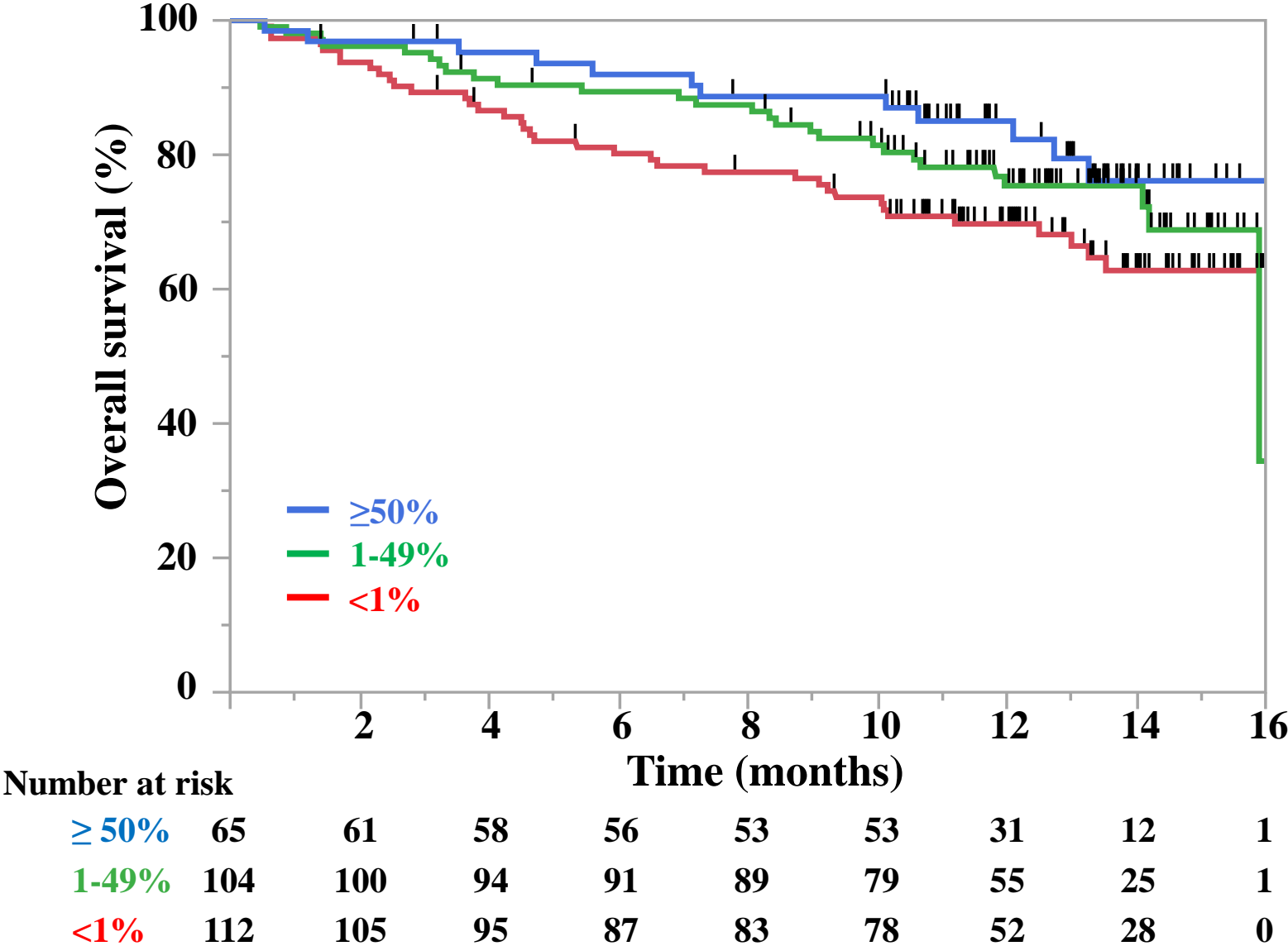

Supplement: Supplemental Data 2 [file mmc2.pdf]
