# Supplementary material for: A Real-World Study on the Effectiveness and Safety of Pembrolizumab Plus Chemotherapy for Nonsquamous NSCLC
Source: JTO Clin Res Rep. 2021 Dec 16;3(2):100265. doi: 10.1016/j.jtocrr.2021.100265 (PMC8819387; doi:10.1016/j.jtocrr.2021.100265)
Supplement: Supplemental Data 3 [file mmc3.docx]

**Supplemental Data 3.** Treatment-related AEs

| Non-hematological AEs, n (%) | Grades 3–5 | Grade 5 |
| --- | --- | --- |
| Any | 57 (19) | 8* (3) |
| Event occurring in ≥3 patients, n |  |  |
| Pneumonitis | 15 | 4 |
| Nephrotoxicity | 3 | – |
| Skin toxicity | 4 | – |
| Colitis | 5 | – |
| Hepatobiliary toxicity** | 10 | – |
| Lung infection | 4 | 1 |
| Nausea | 5 | – |
| Hematological AEs, n (%) | Grades 4–5 | Grade 5 |
| Any | 19 (6) | 2 (1) |
| Event occurring in ≥3 patients, n |  |  |
| Febrile neutropenia | 3 | 2 |
| Neutropenia | 12 | – |
| Anemia | 3 | – |
| Thrombocytopenia | 8 | – |

Abbreviations: AE, adverse event.

* One case of sudden death not otherwise specified and two cases of sepsis.

** Hepatitis in six patients; cholecystitis and cholangitis in one patient each.
