# Supplementary material for: A Real-World Study on the Effectiveness and Safety of Pembrolizumab Plus Chemotherapy for Nonsquamous NSCLC
Source: JTO Clin Res Rep. 2021 Dec 16;3(2):100265. doi: 10.1016/j.jtocrr.2021.100265 (PMC8819387; doi:10.1016/j.jtocrr.2021.100265)
Supplement: Supplemental Data 4 [file mmc4.docx]

**Supplemental Data 4.** Severity of AEs of special interest

| AE, n (%) | Grade 1 | Grade 2 | Grade 3 | Grade 4 | Grade 5 |
| --- | --- | --- | --- | --- | --- |
| Pneumonitis | 17 (5.7) | 22 (7.3) | 9 (3.0) | 2 (0.7) | 4 (1.3) |
| Nephrotoxicity | 45 (15.1) | 13 (4.3) | 2 (0.7) | 1 (0.3) | 0 (0) |

Abbreviations: AE, adverse event.
