# Supplementary material for: A Real-World Study on the Effectiveness and Safety of Pembrolizumab Plus Chemotherapy for Nonsquamous NSCLC
Source: JTO Clin Res Rep. 2021 Dec 16;3(2):100265. doi: 10.1016/j.jtocrr.2021.100265 (PMC8819387; doi:10.1016/j.jtocrr.2021.100265)
Supplement: Supplemental Data 5 [file mmc5.docx]

**Supplemental Data 5.** Patient characteristics and safety profiles stratified by age

| Characteristic | <65 years  (n=110) | 65–74 years  (n=146) | ≥75 years  (n=43) | *p*-value* |
| --- | --- | --- | --- | --- |
| Sex, n (%) |  |  |  | 0.15 |
| Male | 88 (80) | 106 (73) | 28 (65) |  |
| Smoking status, n (%) |  |  |  | <0.001** |
| Current | 61 (55) | 47 (32) | 8 (19) |  |
| Former | 41 (37) | 74 (51) | 19 (44) |  |
| Never | 8 (7) | 25 (17) | 16 (37) |  |
| ECOG PS, n (%) |  |  |  | 0.23** |
| 0 | 36 (33) | 44 (30) | 15 (35) |  |
| 1 | 66 (60) | 96 (66) | 28 (65) |  |
| 2 | 7 (7) | 4 (3) | 0 (0) |  |
| 3 | 1 (1) | 2 (1) | 0 (0) |  |
| Histology, n (%) |  |  |  | 0.053 |
| Adenocarcinoma | 100 (91) | 135 (92) | 43 (100) |  |
| Others | 10 (9) | 11 (8) | 0 (0) |  |
| Stage, n (%) |  |  |  |  |
| 3 | 5 (5) | 4 (3) | 2 (5) |  |
| 4 | 83 (75) | 112 (77) | 33 (77) |  |
| Recurrence after surgery | 14 (13) | 24 (16) | 8 (19) |  |
| Recurrence after radiotherapy | 8 (7) | 6 (4) | 0 (0) |  |
| PD-L1 TPS, n (%) |  |  |  | 0.54** |
| ≥50% | 28 (25) | 30 (21) | 7 (16) |  |
| 1–49% | 38 (35) | 49 (34) | 17 (40) |  |
| <1% | 40 (36) | 57 (39) | 15 (35) |  |
| Not investigated | 4 (4) | 10 (7) | 4 (9) |  |
| Preexisting interstitial lung disease, n (%) | 2 (2) | 4 (3) | 7 (16) | <0.001 |
| Emphysema, n (%) | 40 (36) | 62 (42) | 12 (28) | 0.17 |
| Previous thoracic radiotherapy, n (%) | 16 (15) | 13 (9) | 4 (9) | 1.00 |
|  |  |  |  |  |
| AEs leading to discontinuation of all treatment components | 15 (14) | 39 (27) | 17 (40) | 0.01 |
| Severe AEs | 18 (16) | 31 (21) | 11 (26) | 0.57 |

Abbreviations: AE, adverse event; ECOG PS, Eastern Cooperative Oncology Group performance status; PD-L1, programmed death-ligand 1; TPS, tumor proportion score.

* ≥75 years *versus* <75 years.

** Smokers *versus* never-smokers, ECOG PS 0–1 *versus* ECOG PS 2–3, and ≥50% *versus* <50% PD-L1 expression.
